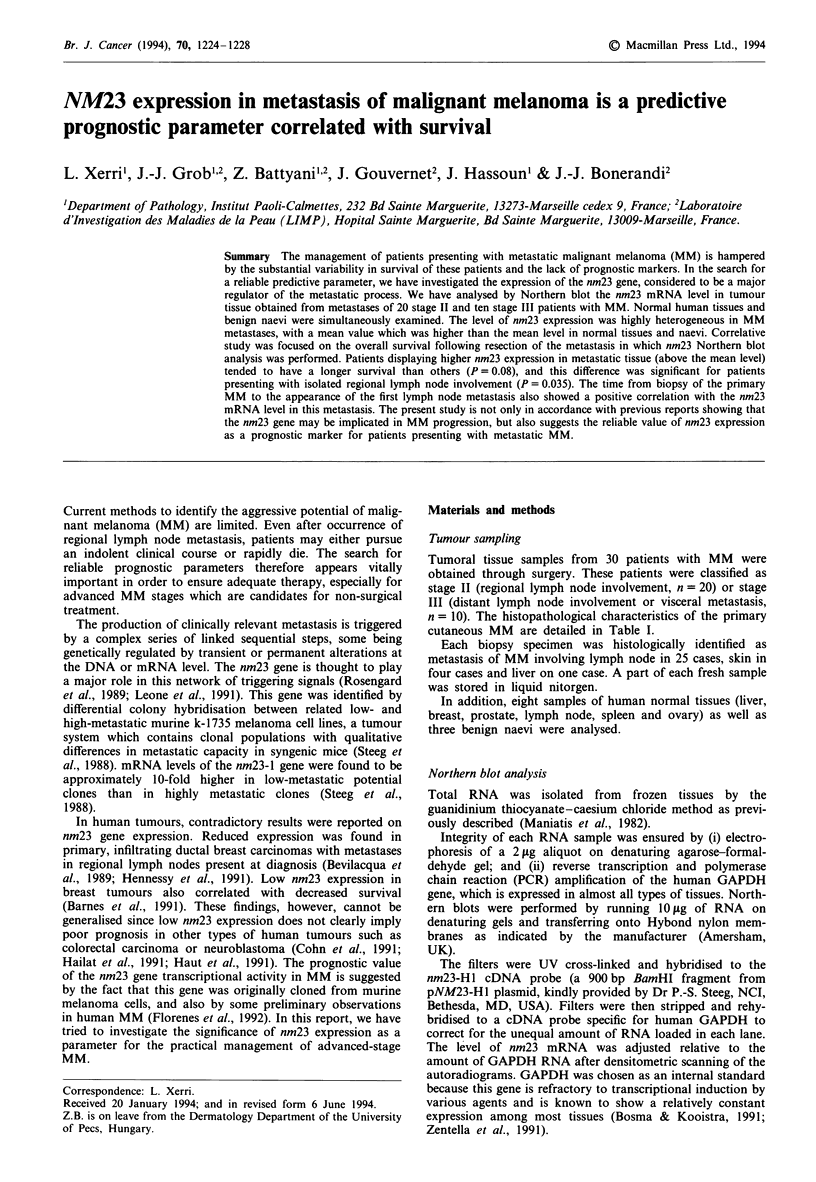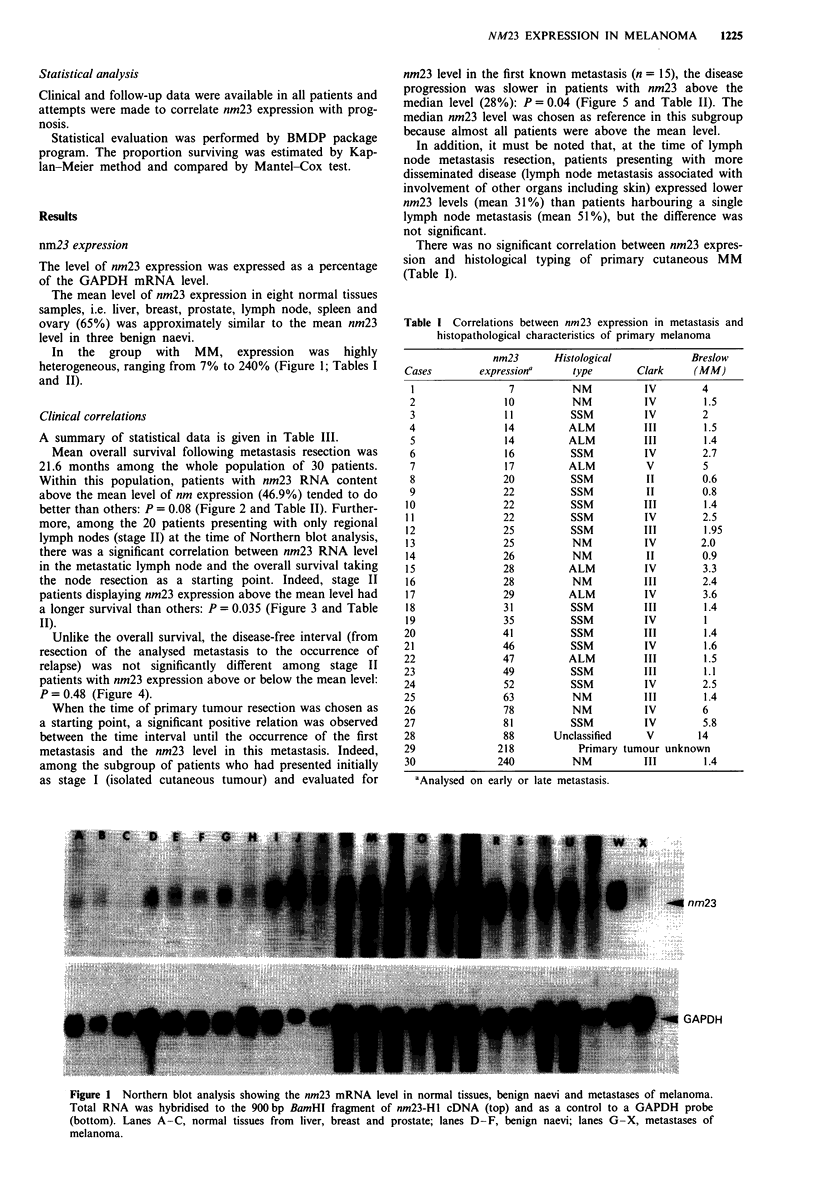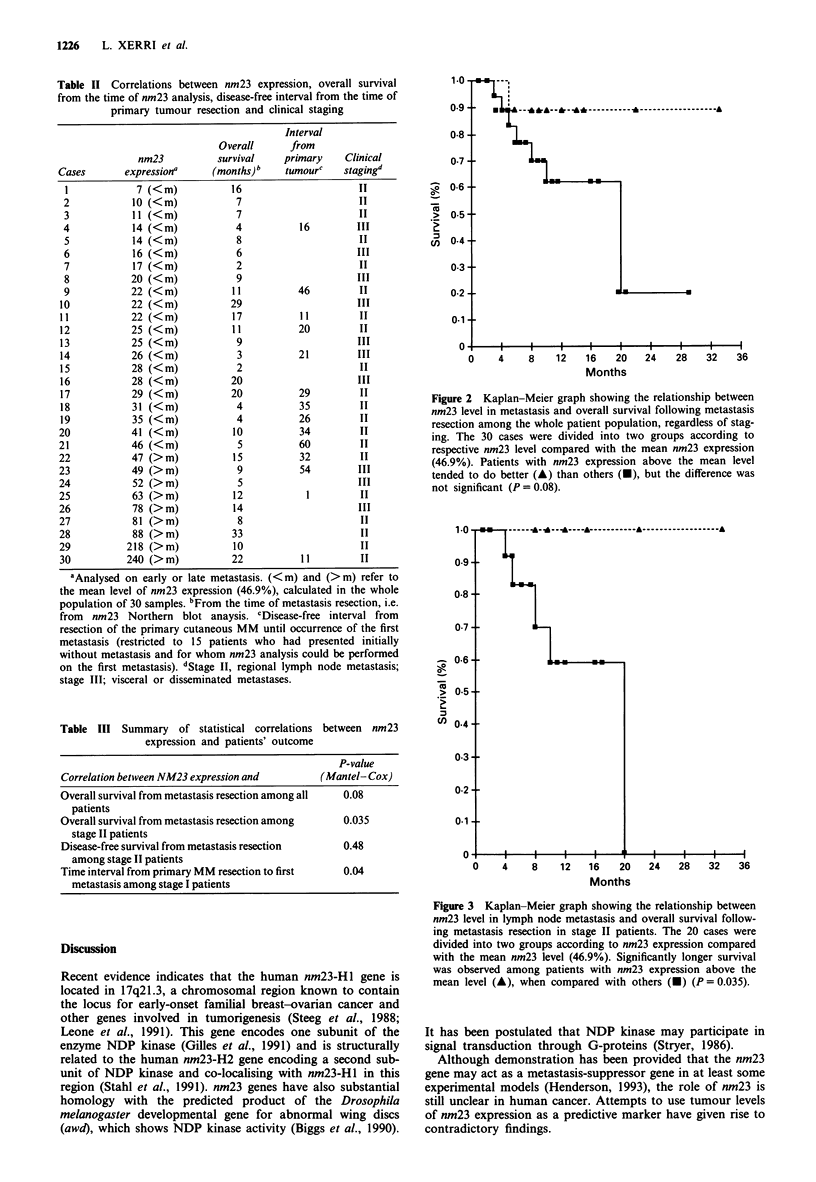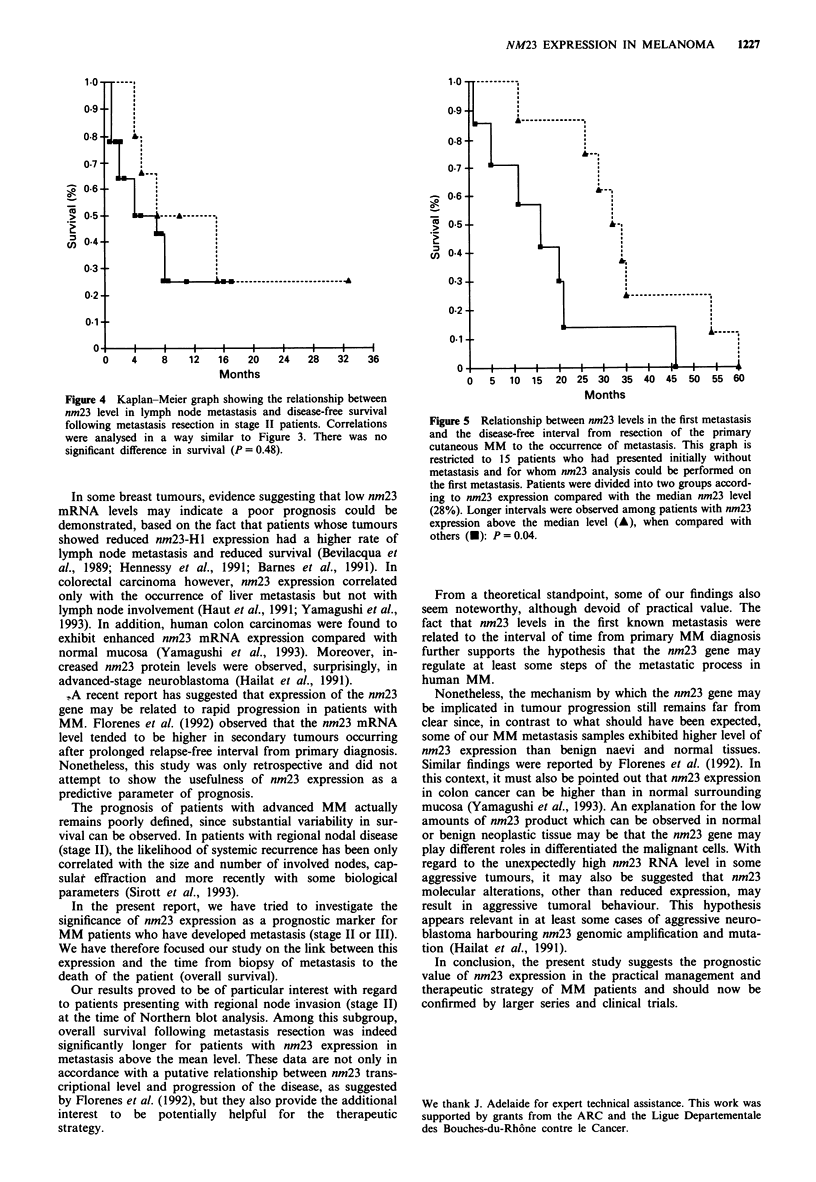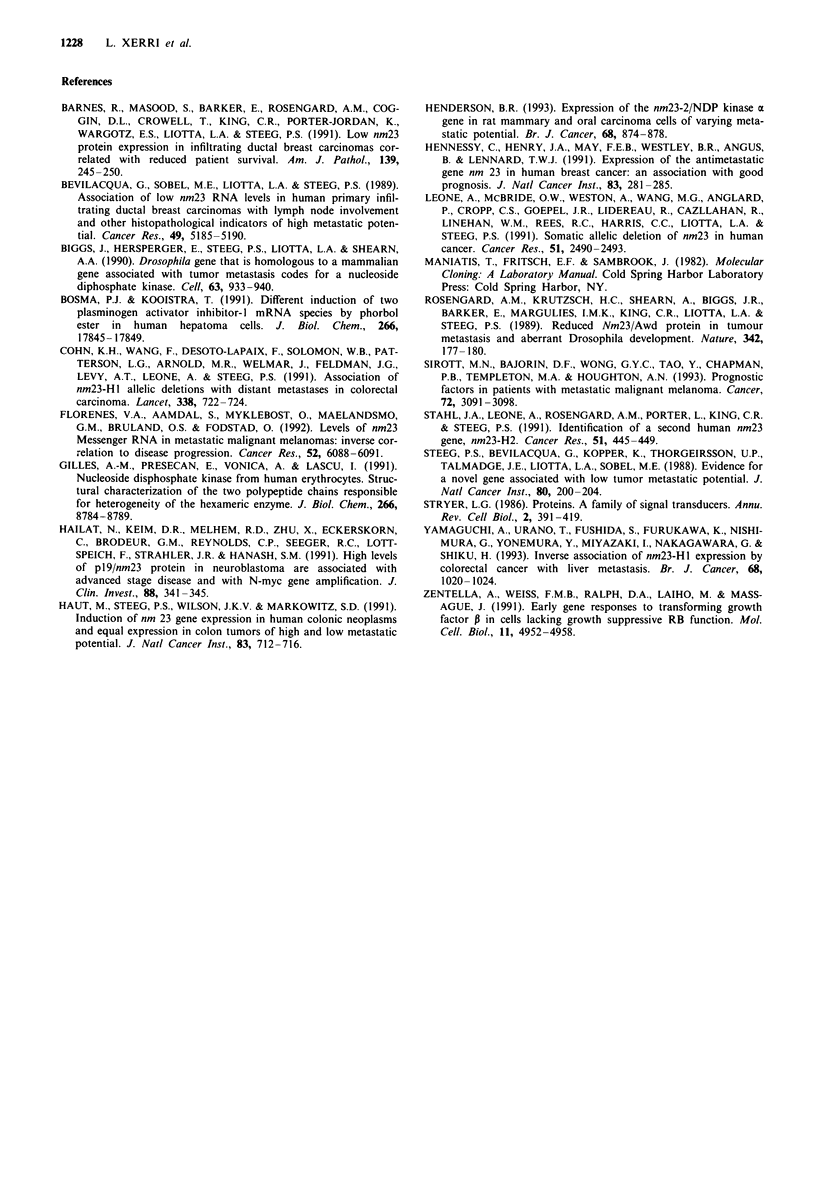# NM23 expression in metastasis of malignant melanoma is a predictive prognostic parameter correlated with survival.

**DOI:** 10.1038/bjc.1994.477

**Published:** 1994-12

**Authors:** L. Xerri, J. J. Grob, Z. Battyani, J. Gouvernet, J. Hassoun, J. J. Bonerandi

**Affiliations:** Department of Pathology, Institut Paoli-Calmettes, Marseille, France.

## Abstract

**Images:**


Br. J. Cancer (1994), 70, 1224–1228
© Macmillan Press Ltd., 1994

### *NM23* expression in metastasis of malignant melanoma is a predictive prognostic parameter correlated with survival

L. Xerri[1], J.-J. Grob[1,2], Z. Battyani[1,2], J. Gouvernet[2], J. Hassoun[1] & J.-J. Bonerandi[2]

[1]*Department of Pathology, Institut Paoli-Calmettes, 232 Bd Sainte Marguerite, 13273-Marseille cedex 9, France;* [2]*Laboratoire d'Investigation des Maladies de la Peau (LIMP), Hopital Sainte Marguerite, Bd Sainte Marguerite, 13009-Marseille, France.*

**Summary** The management of patients presenting with metastatic malignant melanoma (MM) is hampered by the substantial variability in survival of these patients and the lack of prognostic markers. In the search for a reliable predictive parameter, we have investigated the expression of the *nm*23 gene, considered to be a major regulator of the metastatic process. We have analysed by Northern blot the *nm*23 mRNA level in tumour tissue obtained from metastases of 20 stage II and ten stage III patients with MM. Normal human tissues and benign naevi were simultaneously examined. The level of *nm*23 expression was highly heterogeneous in MM metastases, with a mean value which was higher than the mean level in normal tissues and naevi. Correlative study was focused on the overall survival following resection of the metastasis in which *nm*23 Northern blot analysis was performed. Patients displaying higher *nm*23 expression in metastatic tissue (above the mean level) tended to have a longer survival than others ($P = 0.08$), and this difference was significant for patients presenting with isolated regional lymph node involvement ($P = 0.035$). The time from biopsy of the primary MM to the appearance of the first lymph node metastasis also showed a positive correlation with the *nm*23 mRNA level in this metastasis. The present study is not only in accordance with previous reports showing that the *nm*23 gene may be implicated in MM progression, but also suggests the reliable value of *nm*23 expression as a prognostic marker for patients presenting with metastatic MM.

Current methods to identify the aggressive potential of malignant melanoma (MM) are limited. Even after occurrence of regional lymph node metastasis, patients may either pursue an indolent clinical course or rapidly die. The search for reliable prognostic parameters therefore appears vitally important in order to ensure adequate therapy, especially for advanced MM stages which are candidates for non-surgical treatment.

The production of clinically relevant metastasis is triggered by a complex series of linked sequential steps, some being genetically regulated by transient or permanent alterations at the DNA or mRNA level. The *nm*23 gene is thought to play a major role in this network of triggering signals (Rosengard *et al.*, 1989; Leone *et al.*, 1991). This gene was identified by differential colony hybridisation between related low- and high-metastatic murine k-1735 melanoma cell lines, a tumour system which contains clonal populations with qualitative differences in metastatic capacity in syngenic mice (Steeg *et al.*, 1988). mRNA levels of the *nm*23-1 gene were found to be approximately 10-fold higher in low-metastatic potential clones than in highly metastatic clones (Steeg *et al.*, 1988).

In human tumours, contradictory results were reported on *nm*23 gene expression. Reduced expression was found in primary, infiltrating ductal breast carcinomas with metastases in regional lymph nodes present at diagnosis (Bevilacqua *et al.*, 1989; Hennessy *et al.*, 1991). Low *nm*23 expression in breast tumours also correlated with decreased survival (Barnes *et al.*, 1991). These findings, however, cannot be generalised since low *nm*23 expression does not clearly imply poor prognosis in other types of human tumours such as colorectal carcinoma or neuroblastoma (Cohn *et al.*, 1991; Hailat *et al.*, 1991; Haut *et al.*, 1991). The prognostic value of the *nm*23 gene transcriptional activity in MM is suggested by the fact that this gene was originally cloned from murine melanoma cells, and also by some preliminary observations in human MM (Florenes *et al.*, 1992). In this report, we have tried to investigate the significance of *nm*23 expression as a parameter for the practical management of advanced-stage MM.

Correspondence: L. Xerri.
Received 20 January 1994; and in revised form 6 June 1994.
Z.B. is on leave from the Dermatology Department of the University of Pecs, Hungary.

### Materials and methods

### *Tumour sampling*

Tumoral tissue samples from 30 patients with MM were obtained through surgery. These patients were classified as stage II (regional lymph node involvement, $n = 20$) or stage III (distant lymph node involvement or visceral metastasis, $n = 10$). The histopathological characteristics of the primary cutaneous MM are detailed in Table I.

Each biopsy specimen was histologically identified as metastasis of MM involving lymph node in 25 cases, skin in four cases and liver on one case. A part of each fresh sample was stored in liquid nitorgen.

In addition, eight samples of human normal tissues (liver, breast, prostate, lymph node, spleen and ovary) as well as three benign naevi were analysed.

### *Northern blot analysis*

Total RNA was isolated from frozen tissues by the guanidinium thiocyanate–caesium chloride method as previously described (Maniatis *et al.*, 1982).

Integrity of each RNA sample was ensured by (i) electrophoresis of a 2 μg aliquot on denaturing agarose–formaldehyde gel; and (ii) reverse transcription and polymerase chain reaction (PCR) amplification of the human GAPDH gene, which is expressed in almost all types of tissues. Northern blots were performed by running 10 μg of RNA on denaturing gels and transferring onto Hybond nylon membranes as indicated by the manufacturer (Amersham, UK).

The filters were UV cross-linked and hybridised to the *nm*23-H1 cDNA probe (a 900 bp *Bam*HI fragment from p*NM*23-H1 plasmid, kindly provided by Dr P.-S. Steeg, NCI, Bethesda, MD, USA). Filters were then stripped and rehybridised to a cDNA probe specific for human GAPDH to correct for the unequal amount of RNA loaded in each lane. The level of *nm*23 mRNA was adjusted relative to the amount of GAPDH RNA after densitometric scanning of the autoradiograms. GAPDH was chosen as an internal standard because this gene is refractory to transcriptional induction by various agents and is known to show a relatively constant expression among most tissues (Bosma & Kooistra, 1991; Zentella *et al.*, 1991).

*Statistical analysis*

Clinical and follow-up data were available in all patients and attempts were made to correlate *nm*23 expression with prognosis.

Statistical evaluation was performed by BMDP package program. The proportion surviving was estimated by Kaplan–Meier method and compared by Mantel–Cox test.

### Results

*nm23 expression*

The level of *nm*23 expression was expressed as a percentage of the GAPDH mRNA level.

The mean level of *nm*23 expression in eight normal tissues samples, i.e. liver, breast, prostate, lymph node, spleen and ovary (65%) was approximately similar to the mean *nm*23 level in three benign naevi.

In the group with MM, expression was highly heterogeneous, ranging from 7% to 240% (Figure 1; Tables I and II).

*Clinical correlations*

A summary of statistical data is given in Table III.

Mean overall survival following metastasis resection was 21.6 months among the whole population of 30 patients. Within this population, patients with *nm*23 RNA content above the mean level of *nm* expression (46.9%) tended to do better than others: $P = 0.08$ (Figure 2 and Table II). Furthermore, among the 20 patients presenting with only regional lymph nodes (stage II) at the time of Northern blot analysis, there was a significant correlation between *nm*23 RNA level in the metastatic lymph node and the overall survival taking the node resection as a starting point. Indeed, stage II patients displaying *nm*23 expression above the mean level had a longer survival than others: $P = 0.035$ (Figure 3 and Table II).

Unlike the overall survival, the disease-free interval (from resection of the analysed metastasis to the occurrence of relapse) was not significantly different among stage II patients with *nm*23 expression above or below the mean level: $P = 0.48$ (Figure 4).

When the time of primary tumour resection was chosen as a starting point, a significant positive relation was observed between the time interval until the occurrence of the first metastasis and the *nm*23 level in this metastasis. Indeed, among the subgroup of patients who had presented initially as stage I (isolated cutaneous tumour) and evaluated for *nm*23 level in the first known metastasis ($n = 15$), the disease progression was slower in patients with *nm*23 above the median level (28%): $P = 0.04$ (Figure 5 and Table II). The median *nm*23 level was chosen as reference in this subgroup because almost all patients were above the mean level.

In addition, it must be noted that, at the time of lymph node metastasis resection, patients presenting with more disseminated disease (lymph node metastasis associated with involvement of other organs including skin) expressed lower *nm*23 levels (mean 31%) than patients harbouring a single lymph node metastasis (mean 51%), but the difference was not significant.

There was no significant correlation between *nm*23 expression and histological typing of primary cutaneous MM (Table I).

**Table I** Correlations between *nm*23 expression in metastasis and histopathological characteristics of primary melanoma

| *Cases* | *nm23 expression*[a] | *Histological type* | *Clark* | *Breslow (MM)* |
|---|---|---|---|---|
| 1 | 7 | NM | IV | 4 |
| 2 | 10 | NM | IV | 1.5 |
| 3 | 11 | SSM | IV | 2 |
| 4 | 14 | ALM | III | 1.5 |
| 5 | 14 | ALM | III | 1.4 |
| 6 | 16 | SSM | IV | 2.7 |
| 7 | 17 | ALM | V | 5 |
| 8 | 20 | SSM | II | 0.6 |
| 9 | 22 | SSM | II | 0.8 |
| 10 | 22 | SSM | III | 1.4 |
| 11 | 22 | SSM | IV | 2.5 |
| 12 | 25 | SSM | III | 1.95 |
| 13 | 25 | NM | IV | 2.0 |
| 14 | 26 | NM | II | 0.9 |
| 15 | 28 | ALM | IV | 3.3 |
| 16 | 28 | NM | III | 2.4 |
| 17 | 29 | ALM | IV | 3.6 |
| 18 | 31 | SSM | III | 1.4 |
| 19 | 35 | SSM | IV | 1 |
| 20 | 41 | SSM | III | 1.4 |
| 21 | 46 | SSM | IV | 1.6 |
| 22 | 47 | ALM | III | 1.5 |
| 23 | 49 | SSM | III | 1.1 |
| 24 | 52 | SSM | IV | 2.5 |
| 25 | 63 | NM | III | 1.4 |
| 26 | 78 | NM | IV | 6 |
| 27 | 81 | SSM | IV | 5.8 |
| 28 | 88 | Unclassified | V | 14 |
| 29 | 218 | Primary tumour unknown | | |
| 30 | 240 | NM | III | 1.4 |

[a]Analysed on early or late metastasis.

**Figure 1** Northern blot analysis showing the *nm*23 mRNA level in normal tissues, benign naevi and metastases of melanoma. Total RNA was hybridised to the 900 bp *Bam*HI fragment of *nm*23-H1 cDNA (top) and as a control to a GAPDH probe (bottom). Lanes A–C, normal tissues from liver, breast and prostate; lanes D–F, benign naevi; lanes G–X, metastases of melanoma.

**Table II** Correlations between *nm*23 expression, overall survival from the time of *nm*23 analysis, disease-free interval from the time of primary tumour resection and clinical staging

| *Cases* | *nm23 expression*[a] | *Overall survival (months)*[b] | *Interval from primary tumour*[c] | *Clinical staging*[d] |
|---|---|---|---|---|
| 1 | 7 (<m) | 16 | | II |
| 2 | 10 (<m) | 7 | | II |
| 3 | 11 (<m) | 7 | | II |
| 4 | 14 (<m) | 4 | 16 | III |
| 5 | 14 (<m) | 8 | | II |
| 6 | 16 (<m) | 6 | | III |
| 7 | 17 (<m) | 2 | | II |
| 8 | 20 (<m) | 9 | | III |
| 9 | 22 (<m) | 11 | 46 | II |
| 10 | 22 (<m) | 29 | | III |
| 11 | 22 (<m) | 17 | 11 | II |
| 12 | 25 (<m) | 11 | 20 | II |
| 13 | 25 (<m) | 9 | | III |
| 14 | 26 (<m) | 3 | 21 | III |
| 15 | 28 (<m) | 2 | | II |
| 16 | 28 (<m) | 20 | | III |
| 17 | 29 (<m) | 20 | 29 | II |
| 18 | 31 (<m) | 4 | 35 | II |
| 19 | 35 (<m) | 4 | 26 | II |
| 20 | 41 (<m) | 10 | 34 | II |
| 21 | 46 (<m) | 5 | 60 | II |
| 22 | 47 (>m) | 15 | 32 | II |
| 23 | 49 (>m) | 9 | 54 | III |
| 24 | 52 (>m) | 5 | | III |
| 25 | 63 (>m) | 12 | 1 | II |
| 26 | 78 (>m) | 14 | | III |
| 27 | 81 (>m) | 8 | | II |
| 28 | 88 (>m) | 33 | | II |
| 29 | 218 (>m) | 10 | | II |
| 30 | 240 (>m) | 22 | 11 | II |

[a]Analysed on early or late metastasis. (<m) and (>m) refer to the mean level of *nm*23 expression (46.9%), calculated in the whole population of 30 samples. [b]From the time of metastasis resection, i.e. from *nm*23 Northern blot anaysis. [c]Disease-free interval from resection of the primary cutaneous MM until occurrence of the first metastasis (restricted to 15 patients who had presented initially without metastasis and for whom *nm*23 analysis could be performed on the first metastasis). [d]Stage II, regional lymph node metastasis; stage III; visceral or disseminated metastases.

**Table III** Summary of statistical correlations between *nm*23 expression and patients' outcome

| *Correlation between NM23 expression and* | *P-value (Mantel–Cox)* |
|---|---|
| Overall survival from metastasis resection among all patients | 0.08 |
| Overall survival from metastasis resection among stage II patients | 0.035 |
| Disease-free survival from metastasis resection among stage II patients | 0.48 |
| Time interval from primary MM resection to first metastasis among stage I patients | 0.04 |

**Figure 2** Kaplan-Meier graph showing the relationship between *nm*23 level in metastasis and overall survival following metastasis resection among the whole patient population, regardless of staging. The 30 cases were divided into two groups according to respective *nm*23 level compared with the mean *nm*23 expression (46.9%). Patients with *nm*23 expression above the mean level tended to do better (▲) than others (■), but the difference was not significant ($P = 0.08$).

**Figure 3** Kaplan-Meier graph showing the relationship between *nm*23 level in lymph node metastasis and overall survival following metastasis resection in stage II patients. The 20 cases were divided into two groups according to *nm*23 expression compared with the mean *nm*23 level (46.9%). Significantly longer survival was observed among patients with *nm*23 expression above the mean level (▲), when compared with others (■) ($P = 0.035$).

### Discussion

Recent evidence indicates that the human *nm*23-H1 gene is located in 17q21.3, a chromosomal region known to contain the locus for early-onset familial breast–ovarian cancer and other genes involved in tumorigenesis (Steeg *et al.*, 1988; Leone *et al.*, 1991). This gene encodes one subunit of the enzyme NDP kinase (Gilles *et al.*, 1991) and is structurally related to the human *nm*23-H2 gene encoding a second subunit of NDP kinase and co-localising with *nm*23-H1 in this region (Stahl *et al.*, 1991). *nm*23 genes have also substantial homology with the predicted product of the *Drosophila melanogaster* developmental gene for abnormal wing discs (*awd*), which shows NDP kinase activity (Biggs *et al.*, 1990). It has been postulated that NDP kinase may participate in signal transduction through G-proteins (Stryer, 1986).

Although demonstration has been provided that the *nm*23 gene may act as a metastasis-suppressor gene in at least some experimental models (Henderson, 1993), the role of *nm*23 is still unclear in human cancer. Attempts to use tumour levels of *nm*23 expression as a predictive marker have given rise to contradictory findings.

**Figure 4** Kaplan–Meier graph showing the relationship between *nm*23 level in lymph node metastasis and disease-free survival following metastasis resection in stage II patients. Correlations were analysed in a way similar to Figure 3. There was no significant difference in survival ($P = 0.48$).

In some breast tumours, evidence suggesting that low *nm*23 mRNA levels may indicate a poor prognosis could be demonstrated, based on the fact that patients whose tumours showed reduced *nm*23-H1 expression had a higher rate of lymph node metastasis and reduced survival (Bevilacqua *et al.*, 1989; Hennessy *et al.*, 1991; Barnes *et al.*, 1991). In colorectal carcinoma however, *nm*23 expression correlated only with the occurrence of liver metastasis but not with lymph node involvement (Haut *et al.*, 1991; Yamagushi *et al.*, 1993). In addition, human colon carcinomas were found to exhibit enhanced *nm*23 mRNA expression compared with normal mucosa (Yamagushi *et al.*, 1993). Moreover, increased *nm*23 protein levels were observed, surprisingly, in advanced-stage neuroblastoma (Hailat *et al.*, 1991).

A recent report has suggested that expression of the *nm*23 gene may be related to rapid progression in patients with MM. Florenes *et al.* (1992) observed that the *nm*23 mRNA level tended to be higher in secondary tumours occurring after prolonged relapse-free interval from primary diagnosis. Nonetheless, this study was only retrospective and did not attempt to show the usefulness of *nm*23 expression as a predictive parameter of prognosis.

The prognosis of patients with advanced MM actually remains poorly defined, since substantial variability in survival can be observed. In patients with regional nodal disease (stage II), the likelihood of systemic recurrence has been only correlated with the size and number of involved nodes, capsular effraction and more recently with some biological parameters (Sirott *et al.*, 1993).

In the present report, we have tried to investigate the significance of *nm*23 expression as a prognostic marker for MM patients who have developed metastasis (stage II or III). We have therefore focused our study on the link between this expression and the time from biopsy of metastasis to the death of the patient (overall survival).

Our results proved to be of particular interest with regard to patients presenting with regional node invasion (stage II) at the time of Northern blot analysis. Among this subgroup, overall survival following metastasis resection was indeed significantly longer for patients with *nm*23 expression in metastasis above the mean level. These data are not only in accordance with a putative relationship between *nm*23 transcriptional level and progression of the disease, as suggested by Florenes *et al.* (1992), but they also provide the additional interest to be potentially helpful for the therapeutic strategy.

**Figure 5** Relationship between *nm*23 levels in the first metastasis and the disease-free interval from resection of the primary cutaneous MM to the occurrence of metastasis. This graph is restricted to 15 patients who had presented initially without metastasis and for whom *nm*23 analysis could be performed on the first metastasis. Patients were divided into two groups according to *nm*23 expression compared with the median *nm*23 level (28%). Longer intervals were observed among patients with *nm*23 expression above the median level (▲), when compared with others (■): $P = 0.04$.

From a theoretical standpoint, some of our findings also seem noteworthy, although devoid of practical value. The fact that *nm*23 levels in the first known metastasis were related to the interval of time from primary MM diagnosis further supports the hypothesis that the *nm*23 gene may regulate at least some steps of the metastatic process in human MM.

Nonetheless, the mechanism by which the *nm*23 gene may be implicated in tumour progression still remains far from clear since, in contrast to what should have been expected, some of our MM metastasis samples exhibited higher level of *nm*23 expression than benign naevi and normal tissues. Similar findings were reported by Florenes *et al.* (1992). In this context, it must also be pointed out that *nm*23 expression in colon cancer can be higher than in normal surrounding mucosa (Yamagushi *et al.*, 1993). An explanation for the low amounts of *nm*23 product which can be observed in normal or benign neoplastic tissue may be that the *nm*23 gene may play different roles in differentiated the malignant cells. With regard to the unexpectedly high *nm*23 RNA level in some aggressive tumours, it may also be suggested that *nm*23 molecular alterations, other than reduced expression, may result in aggressive tumoral behaviour. This hypothesis appears relevant in at least some cases of aggressive neuroblastoma harbouring *nm*23 genomic amplification and mutation (Hailat *et al.*, 1991).

In conclusion, the present study suggests the prognostic value of *nm*23 expression in the practical management and therapeutic strategy of MM patients and should now be confirmed by larger series and clinical trials.

We thank J. Adelaide for expert technical assistance. This work was supported by grants from the ARC and the Ligue Departementale des Bouches-du-Rhône contre le Cancer.

### References

BARNES, R., MASOOD, S., BARKER, E., ROSENGARD, A.M., COGGIN, D.L., CROWELL, T., KING, C.R., PORTER-JORDAN, K., WARGOTZ, E.S., LIOTTA, L.A. & STEEG, P.S. (1991). Low *nm*23 protein expression in infiltrating ductal breast carcinomas correlated with reduced patient survival. *Am. J. Pathol.*, **139**, 245-250.

BEVILACQUA, G., SOBEL, M.E., LIOTTA, L.A. & STEEG, P.S. (1989). Association of low *nm*23 RNA levels in human primary infiltrating ductal breast carcinomas with lymph node involvement and other histopathological indicators of high metastatic potential. *Cancer Res.*, **49**, 5185-5190.

BIGGS, J., HERSPERGER, E., STEEG, P.S., LIOTTA, L.A. & SHEARN, A.A. (1990). *Drosophila* gene that is homologous to a mammalian gene associated with tumor metastasis codes for a nucleoside diphosphate kinase. *Cell*, **63**, 933-940.

BOSMA, P.J. & KOOISTRA, T. (1991). Different induction of two plasminogen activator inhibitor-1 mRNA species by phorbol ester in human hepatoma cells. *J. Biol. Chem.*, **266**, 17845-17849.

COHN, K.H., WANG, F., DESOTO-LAPAIX, F., SOLOMON, W.B., PATTERSON, L.G., ARNOLD, M.R., WELMAR, J., FELDMAN, J.G., LEVY, A.T., LEONE, A. & STEEG, P.S. (1991). Association of *nm*23-H1 allelic deletions with distant metastases in colorectal carcinoma. *Lancet*, **338**, 722-724.

FLORENES, V.A., AAMDAL, S., MYKLEBOST, O., MAELANDSMO, G.M., BRULAND, O.S. & FODSTAD, O. (1992). Levels of *nm*23 Messenger RNA in metastatic malignant melanomas: inverse correlation to disease progression. *Cancer Res.*, **52**, 6088-6091.

GILLES, A.-M., PRESECAN, E., VONICA, A. & LASCU, I. (1991). Nucleoside disphosphate kinase from human erythrocytes. Structural characterization of the two polypeptide chains responsible for heterogeneity of the hexameric enzyme. *J. Biol. Chem.*, **266**, 8784-8789.

HAILAT, N., KEIM, D.R., MELHEM, R.D., ZHU, X., ECKERSKORN, C., BRODEUR, G.M., REYNOLDS, C.P., SEEGER, R.C., LOTTSPEICH, F., STRAHLER, J.R. & HANASH, S.M. (1991). High levels of p19/*nm*23 protein in neuroblastoma are associated with advanced stage disease and with N-myc gene amplification. *J. Clin. Invest.*, **88**, 341-345.

HAUT, M., STEEG, P.S., WILSON, J.K.V. & MARKOWITZ, S.D. (1991). Induction of *nm* 23 gene expression in human colonic neoplasms and equal expression in colon tumors of high and low metastatic potential. *J. Natl Cancer Inst.*, **83**, 712-716.

HENDERSON, B.R. (1993). Expression of the *nm*23-2/NDP kinase α gene in rat mammary and oral carcinoma cells of varying metastatic potential. *Br. J. Cancer*, **68**, 874-878.

HENNESSY, C., HENRY, J.A., MAY, F.E.B., WESTLEY, B.R., ANGUS, B. & LENNARD, T.W.J. (1991). Expression of the antimetastatic gene *nm* 23 in human breast cancer: an association with good prognosis. *J. Natl Cancer Inst.*, **83**, 281-285.

LEONE, A., MCBRIDE, O.W., WESTON, A., WANG, M.G., ANGLARD, P., CROPP, C.S., GOEPEL, J.R., LIDEREAU, R., CAZLLAHAN, R., LINEHAN, W.M., REES, R.C., HARRIS, C.C., LIOTTA, L.A. & STEEG, P.S. (1991). Somatic allelic deletion of *nm*23 in human cancer. *Cancer Res.*, **51**, 2490-2493.

MANIATIS, T., FRITSCH, E.F. & SAMBROOK, J. (1982). *Molecular Cloning: A Laboratory Manual.* Cold Spring Harbor Laboratory Press: Cold Spring Harbor, NY.

ROSENGARD, A.M., KRUTZSCH, H.C., SHEARN, A., BIGGS, J.R., BARKER, E., MARGULIES, I.M.K., KING, C.R., LIOTTA, L.A. & STEEG, P.S. (1989). Reduced *Nm*23/Awd protein in tumour metastasis and aberrant Drosophila development. *Nature*, **342**, 177-180.

SIROTT, M.N., BAJORIN, D.F., WONG, G.Y.C., TAO, Y., CHAPMAN, P.B., TEMPLETON, M.A. & HOUGHTON, A.N. (1993). Prognostic factors in patients with metastatic malignant melanoma. *Cancer*, **72**, 3091-3098.

STAHL, J.A., LEONE, A., ROSENGARD, A.M., PORTER, L., KING, C.R. & STEEG, P.S. (1991). Identification of a second human *nm*23 gene, *nm*23-H2. *Cancer Res.*, **51**, 445-449.

STEEG, P.S., BEVILACQUA, G., KOPPER, K., THORGEIRSSON, U.P., TALMADGE, J.E., LIOTTA, L.A., SOBEL, M.E. (1988). Evidence for a novel gene associated with low tumor metastatic potential. *J. Natl Cancer Inst.*, **80**, 200-204.

STRYER, L.G. (1986). Proteins. A family of signal transducers. *Annu. Rev. Cell Biol.*, **2**, 391-419.

YAMAGUCHI, A., URANO, T., FUSHIDA, S., FURUKAWA, K., NISHIMURA, G., YONEMURA, Y., MIYAZAKI, I., NAKAGAWARA, G. & SHIKU, H. (1993). Inverse association of *nm*23-H1 expression by colorectal cancer with liver metastasis. *Br. J. Cancer*, **68**, 1020-1024.

ZENTELLA, A., WEISS, F.M.B., RALPH, D.A., LAIHO, M. & MASSAGUE, J. (1991). Early gene responses to transforming growth factor β in cells lacking growth suppressive RB function. *Mol. Cell. Biol.*, **11**, 4952-4958.